# The Paradoxical Effect of Cannabis Use on Cognition in Chronic Psychotic Disorders

**DOI:** 10.3390/pathophysiology33010011

**Published:** 2026-01-27

**Authors:** Fiorela Gorea, Martina Pelle, Federico Fiori Nastro, Carmine Gelormini, Fatime Elezi, Michele Ribolsi, Giorgio Di Lorenzo

**Affiliations:** 1 Psychiatry Department, “Mother Teresa” University Hospital Center, 1001 Tirana, Albania; fiorelagorea@yahoo.com (F.G.); fata_el@yahoo.it (F.E.); 2 Unit of Neurology, Neurophysiology, Neurobiology and Psychiatry, Department of Medicine, Campus Bio-Medico University, 00128 Rome, Italy; m.pelle@policlinicocampus.it; 3 Chair of Psychiatry, Department of Systems Medicine, Tor Vergata University of Rome, 00133 Rome, Italy; federico.fiori.nastro@uniroma2.it (F.F.N.); di.lorenzo@med.uniroma2.it (G.D.L.); 4 IRCCS Fondazione Santa Lucia, 00179 Rome, Italy; 5 Institute of Biomedical and Neural Engineering, Reykjavik University, 101 Reykjavik, Iceland; carmine23@ru.is; 6 Chair of Psychiatry, Department of Life Science, Health, and Health Professions, Link Campus University, 00165 Rome, Italy

**Keywords:** cannabis, psychosis, first-episode psychosis, schizophrenia, induced psychosis, adolescents, cognition, MoCA

## Abstract

Background/Objectives: Cannabis use has a particularly high prevalence in individuals with psychotic disorders. Although cannabis use is generally associated with cognitive impairments in the general population, its impact on cognition in psychosis remains controversial. This study aimed to investigate the association between cannabis use and cognitive performance in a cohort of individuals affected by psychotic disorders. Methods: A total of 105 inpatients with psychotic disorders (mean age: 40.3 years; 34 females) were recruited from the University Hospital Center “Mother Teresa” in Tirana. Data collection included socio-demographic and clinical variables. Cognitive functioning was evaluated using the Montreal Cognitive Assessment (MoCA), while psychopathology was assessed with the Brief Negative Symptom Scale (BNSS), the Calgary Depression Scale for Schizophrenia (CDSS), the Psychotic Symptom Rating Scales (PSYRATS), and the Scale for the Assessment of Thought, Language, and Communication (TLC). Results: Cannabis users (CU) were more frequently male, younger, and exhibited an earlier onset of psychosis compared to non-users (No-CU). Importantly, CU demonstrated higher MoCA scores, with the most favorable outcomes observed among daily users. Conclusions: Contrary to the prevailing assumption that cannabis use exacerbates cognitive decline, our findings indicate an unexpected association between cannabis use and preserved cognitive functioning in psychosis. These results underscore the need to consider dosage, frequency, and cannabinoid composition (THC/CBD ratio) when interpreting cannabis-related cognitive outcomes in psychotic disorders.

## 1. Introduction

Cannabis is one of the most widely used recreational psychoactive substances among adolescents and young adults, according to the World Health Organization [1,2], with particularly high prevalence reported in individuals with psychosis [3]. The increasing popularity of cannabis has been accompanied by increasing public concern about its safety. A growing body of evidence links cannabis use to adverse behavioral, physiological, and neural effects [4], with findings suggesting a potential causal relationship [5]. Nonetheless, the harmful effects of cannabis remain a subject of debate, particularly regarding their severity and potential long-term persistence.

Cannabis is the most widely used illicit drug in Europe, with approximately 8–9% of adults aged 15–64 reporting cannabis use in the past year [6]; however, prevalence rates increase to about 27% among individuals with schizophrenia (SCZ) and reach up to 65% in those experiencing a first-episode psychosis (FEP) [7]. The relationship between substance use and psychosis is bidirectional; individuals with psychosis may consume substances as a coping mechanism to alleviate symptoms or side effects, while cannabis use itself can precipitate or exacerbate both attenuated and acute psychotic symptoms [8].

To date, more than 60 cannabinoids have been identified in cannabis, of which Δ9-tetrahydrocannabinol (THC) and cannabidiol (CBD) are the most pharmacologically active [9]. Cannabinoids act mainly through CB1 and CB2 receptors of the endocannabinoid system, modulating GABA and glutamate release, and thereby influencing broader neurotransmitter systems, including dopamine, acetylcholine, and norepinephrine [10]. These mechanisms underlie the effects of cannabis on reward processing, learning, and pain perception [11]. Cognitive functioning has been one of the most extensively studied domains in relation to cannabis use; however, it remains unclear whether cannabis use is linked to persistent neuropsychological deficits [12].

Neurocognitive deficits are a core feature of psychotic disorders, with significant implications for illness course and prognosis [13]. Cognitive impairment contributes substantially to the direct and indirect costs of SCZ [14], undermines treatment adherence, and increases the risk of relapse. These impairments often lead to reduced self-care, greater reliance on inpatient and outpatient services, and lower productivity for both patients and caregivers [15]. Notably, cannabis-related cognitive and functional impairments in SCZ appear to develop early and remain relatively stable throughout the illness course [16].

Although cannabis use has been associated with an elevated risk of psychotic-spectrum symptoms [17], its impact on cognition and brain function remains controversial [7]. SCZ is associated with robust deficits across multiple cognitive domains, including verbal abilities [18], memory [19], attention [20], processing speed [21], and executive functions [22]. In non-psychotic subjects, regular and heavy cannabis use is linked to mild cognitive deficits and to structural and functional brain alterations [23]. Interestingly, some studies have reported that patients with a history of cannabis use prior to the onset of psychosis may present with fewer attentional deficits compared with non-users [24,25,26]. Furthermore, preliminary evidence suggests that CBD may have therapeutic effects, potentially improving some cognitive and motivational deficits [27].

Two meta-analyses [28,29] reported that cannabis users perform worse than non-users across overall neuropsychological measures. Domains most affected included executive functions, attention, learning and memory, motor skills, and verbal abilities, although effect sizes were modest (about one-third of a standard deviation). Notably, Schreiner and colleagues [29] found no significant cognitive differences between users and non-users following at least one month of abstinence, suggesting partial reversibility of cannabis-related cognitive deficits. The severity and persistence of these impairments appear to depend on frequency and duration of use, abstinence length, and age of onset, with adolescence representing a particularly vulnerable developmental stage [30].

Several neuropsychological tools have been developed to assess cognitive deficits in psychotic disorders, including the MATRICS Consensus Cognitive Battery (MCCB) [31,32], the Brief Assessment of Cognition in Schizophrenia (BACS) [33], the Screen for Cognitive Impairment in Psychiatry (SCIP) [34], and the Brief Neurocognitive Assessment (BNA) [35]. Although valid, these instruments are often time-consuming, require specialized training, or target limited cognitive domains, limiting their feasibility in routine clinical practice [35].

The Montreal Cognitive Assessment (MoCA) [36] is a brief, sensitive screening tool that requires only 10–15 min to administer and demonstrates strong validity in detecting cognitive impairment across various conditions [37,38]. Originally developed to identify mild cognitive impairment (MCI), MoCA has also proven effective in identifying deficits in individuals with early psychosis, including individuals with FEP, clinical high risk of psychosis (CHR-P) state, and Psychotic-like Experiences (PLE) [39].

The aim of the present study is to evaluate neurocognitive deficits in individuals with affective and non-affective psychosis using the MoCA and to investigate the relationship between cognitive impairment and cannabis use in this population.

## 2. Materials and Methods

### 2.1. Participants

This study included a convenience sample of one hundred and five Albanian patients diagnosed with a psychotic spectrum disorder. Participants were recruited from the University Hospital Center “Mother Teresa” in Tirana between January 2022 and January 2023. All participants were hospitalized at the time of recruitment and had a history of multiple previous hospitalizations. Diagnoses were made according to DSM-5 criteria [40].

The adopted inclusion criteria for participation in the study were a confirmed diagnosis of affective or non-affective psychosis, based on standardized clinical assessment, and the provision of written

Informed consent by the participant. The adopted exclusion criteria were age under 18 or over 60 years old and an intelligence quotient equal to or less than 70, established by the Wechsler Adult Intelligence Scale-Revised (WAIS-R) [41].

Patients were given the opportunity to ask questions prior to completing clinician-administered questionnaires. No incentives were offered for participation. Anonymity and confidentiality of personal data were guaranteed.

The study was approved by the Independent Ethics Committee of Policlinico Tor Vergata (#184/25; 26 June 2025), and informed consent was obtained from all participants.

### 2.2. Data Collection

Socio-demographic data (e.g., age, sex, marital status) and clinical information (e.g., alcohol and cannabis use, years since diagnosis, number of hospitalizations, family history of mental health disorders, current therapy, and use of long-acting antipsychotics) were collected through structured clinical interviews and medical records.

### 2.3. Assessment

All participants underwent a comprehensive clinical assessment, including psychopathological and cognitive evaluations.

#### 2.3.1. Psychopathological Measures

The Brief Negative Symptom Scale (BNSS) is a 13-item semi-structured scale that assesses six negative symptom domains: anhedonia, distress, asociality, avolition, blunted affect, and alogia [42,43]. Items are rated on a Likert 7-point scale (0–6), with higher scores indicating greater symptom severity. The total score ranges from 0 to 78. Two factors can be derived: Motivation and Pleasure (MAP) (summing anhedonia, avolition, and asociality) and Emotional Expressivity (EE) (summing blunted affect and alogia).

The Calgary Depression Scale for Schizophrenia (CDSS) is a 9-item scale measuring depressive symptoms in subjects with SCZ, including depression, hopelessness, guilt, and suicidal ideation [44]. Items are scored on a 4-point scale (0–3), resulting in a total score ranging from 0 to 27. A score higher than 6 suggests a high probability of a major depressive episode.

The Psychotic Symptom Rating Scales (PSYRATS) are semi-structured interviews assessing the severity and subjective characteristics of hallucinations and delusions [45]. The Auditory Hallucinations Subscale (AHS) includes 11 items (e.g., frequency, controllability, and negative content), while the Delusions Subscale (DS) includes 6 items (e.g., preoccupation and intensity of disturbance).

The Thought, Language, and Communication (TLC) scale assesses formal thought disorder (FTD) and language disturbances [46]. It categorizes 18 types of language disruptions into “more pathological” (e.g., derailment, incoherence, clanging, neologisms) and “less pathological” (e.g., circumstantiality, perseveration, blocking). Severity scores are weighted by pathology level and summed to yield a total score.

#### 2.3.2. Translation of Psychopathological Instruments

To ensure cultural and linguistic validity, all psychopathological instruments (BNSS, CDSS, PSYRATS, and TLC) were translated into Albanian following established guidelines. Two professional translators independently produced Albanian versions, which were synthesized into a single harmonized version. This version was then back-translated into English by a third professional translator. The back-translations were compared to the original instruments, and any discrepancies were resolved. The final versions were reviewed by a panel of psychiatrists, clinical psychologists, and translators to ensure cultural appropriateness and linguistic accuracy. Pilot testing was conducted in a subsample of the target population, and feedback was incorporated to finalize the instruments.

#### 2.3.3. Cognitive Assessment

The Montreal Cognitive Assessment (MoCA) evaluates multiple cognitive domains, including attention, executive functioning, memory, language, visuoconstruction, conceptual thinking, calculations, and orientation [37]. Administration takes approximately 10 min. Total scores range from 0 to 30, with a cut-off of ≥26 indicating normal cognitive performance.

#### 2.3.4. Cannabis Use Assessment

Cannabis use was assessed through structured clinical interviews. Participants were classified using both quantitative and frequency-based measures. Based on cannabis use, two groups were identified: cannabis users (CU) and non-users (No-CU), with the No-CU group exclusively including individuals with no lifetime history of cannabis use. The frequency-based approach retained the non-user group (No-CU) and further subdivided CU into low-frequency users (L-CU), defined as sporadic use occurring at least three times per month, and high-frequency users (H-CU), referring to patients who reported daily cannabis consumption.

### 2.4. Statistical Analysis

Statistical analysis was performed using R (version 4.5.2) [47]. Group differences were evaluated using non-parametric methods. This approach was preferred due to its robustness to violations of normality and homogeneity of variance assumptions.

First, differences in sociodemographic and clinical characteristics were explored between CU and No-CU. Then, continuous cognitive variables were compared between the CU and No-CU groups using the Mann–Whitney U test (also known as the Wilcoxon rank-sum test). The analyses were conducted using the wilcox_test() function from the coin package in R. For each MoCA variable, distributions were compared between groups using an approximate permutation distribution based on 10,000 resamples to improve the precision of *p*-value estimation. From each test, Z statistics and raw *p*-values were extracted. To adjust for multiple comparisons across variables, the False Discovery Rate (FDR) correction (Benjamini–Hochberg procedure) was applied, and statistical significance was set at *p* < 0.05 after correction.

Moreover, a further analysis was conducted to compare the three independent groups, defined according to cannabis use level (No-CU, L-CU, H-CU), using the Kruskal–Wallis test implemented via the kruskal_test() function in the coin package with 10,000 permutation resamples. For each variable, the test statistic (H, approximately chi-squared distributed) and *p*-values were obtained. To adjust for multiple comparisons across variables, the FDR correction (Benjamini–Hochberg procedure) was applied, and statistical significance was set at *p* < 0.05 after correction. When significant omnibus effects were detected, post hoc pairwise comparisons were conducted using the Dwass–Steel–Critchlow–Fligner (DSCF) test, with *p*-values adjusted for multiple testing.

## 3. Results

One hundred and five subjects (34 females, 32.3%; age range, 20–60 years; mean ± SD, 40.3 ± 11.8) were enrolled in the study.

Differences in sex, age, and psychosis onset were highlighted, with CU participants significantly more likely to be male (*p* = 0.004), younger (*p* < 0.001), and to have an earlier onset of psychosis (*p* < 0.001) compared to No-CU.

With regard to psychopathology, CU showed significantly lower impairments in the Motivation and Pleasure (MAP) subdomain of BNSS compared to No-CU (*p* = 0.011). However, no other significant differences in psychopathological measures were observed between the two groups. Sociodemographic and psychopathological descriptive and inferential analyses are presented in Table 1.

Mann–Whitney U tests revealed significant differences between CU and No-CU on several MoCA measures. No-CU showed significantly lower scores on the overall MoCA (Z = −3.70, *p* = 0.0003, FDR-adjusted *p* = 0.0024), the Visuospatial/Executive subscale (Z = −2.50, *p* = 0.0142, FDR *p* = 0.0227), the Attention subscale (Z = −2.85, *p* = 0.0039, FDR *p* = 0.0104), the Abstraction subscale (Z = −3.21, *p* = 0.0006, FDR *p* = 0.0024), and the Orientation subscale (Z = −2.80, *p* = 0.0063, FDR *p* = 0.0126). The distribution of the data indicates that CU showed higher scores across all MoCA variables except for Delayed Recall. Nevertheless, no statistically significant differences emerged for the Naming, Language, or Delayed Recall subscales (*p* > 0.05 after FDR correction) (Figure S1). Cognitive differences between CU and No-CU are presented in Table 2.

Kruskal–Wallis tests indicated significant group effects on overall MoCA performance (H(2) = 15.05, *p* = 0.0003, FDR *p* = 0.0024), as well as for the Visuospatial/Executive (H(2) = 8.75, *p* = 0.0102, FDR *p* = 0.0262), Attention (H(2) = 8.15, *p* = 0.0154, FDR *p* = 0.0262), Abstraction (H(2) = 11.42, *p* = 0.0029, FDR *p* = 0.0116), and Orientation (H(2) = 7.98, *p* = 0.0164, FDR *p* = 0.0262) subscales.

Post hoc Dwass–Steel–Critchlow–Fligner tests revealed that No-CU scored significantly lower than both L-CU and H-CU on the overall MoCA score, Abstraction, and lower than L-CU in Orientation scales (all *p* < 0.05). For Visuospatial/Executive, No-CU performed significantly worse than H-CU. As previously mentioned, the distribution of the data indicates that participants with H-CU have higher scores than those with No-CU. No significant pairwise differences emerged for Naming, Language, or Delayed Recall. Cognitive differences among No-CU, L-CU, and H-CU are presented in Table 3 and Table 4 (Figure S2).

**Figure 1:**
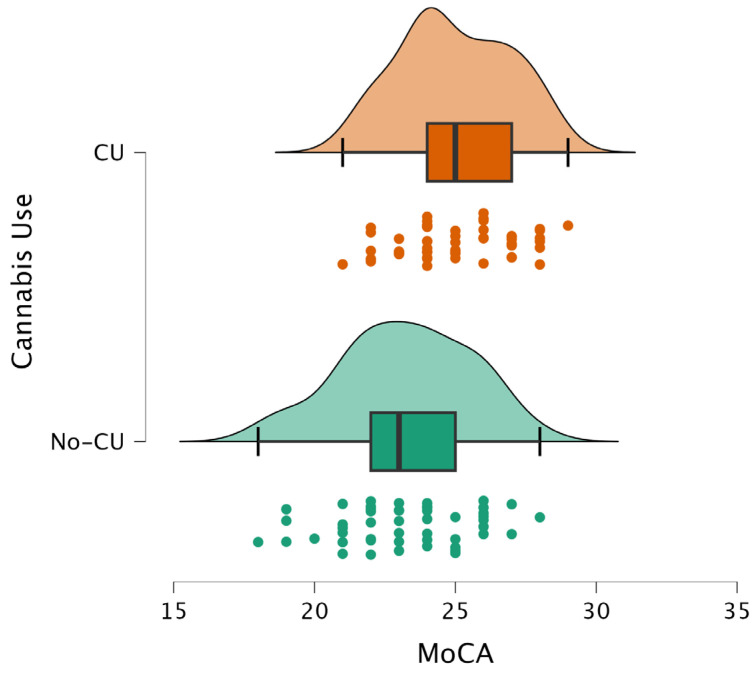
Distribution of MoCA scores in cannabis users (CU) and non-users (No-CU). CU shows a higher median MoCA score compared to the No-CU group.

**Figure 2:**
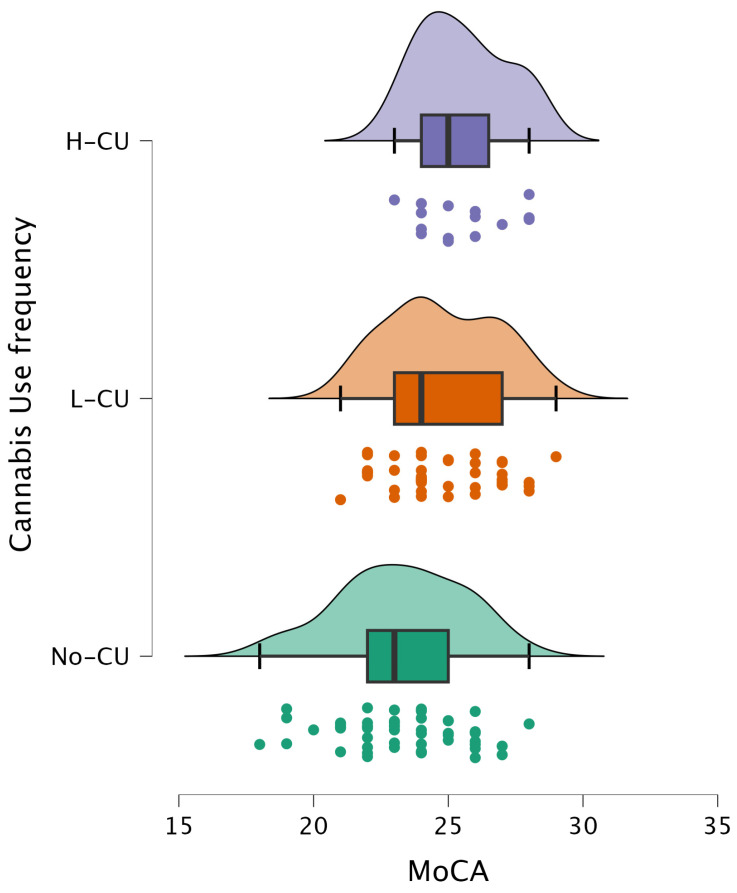
Distribution of MoCA scores across cannabis use frequency groups. Participants were categorized as non-users (No-CU), low-frequency users (L-CU), and high-frequency users (H-CU).

Cognitive differences are also illustrated in Figure 1 and Figure 2.

## 4. Discussion

Cannabis use is commonly associated with cognitive impairment, with robust evidence documenting deficits in memory, attention, and executive functioning across both recreational and clinical populations [48,49,50]. In individuals with psychotic disorders, cannabis use is typically associated with poorer long-term outcomes, increased relapse rates, reduced treatment adherence, and more severe symptom trajectories [30,51].

However, our findings point to a more complex and paradoxical association between cannabis use and cognition in individuals with psychosis.

The CU group displayed significantly higher global cognitive performance compared to the No-CU group, as reflected by MoCA total scores and multiple domain-specific indices. These differences remained robust after permutation-based inference and FDR correction, and the distribution of the data consistently showed higher scores among cannabis-using patients. No differences emerged in Naming, Language, or Delayed Recall subscales.

This result is consistent with emerging evidence that challenges the uniform assumption of cannabis-related cognitive decline in psychosis. Several studies have reported that cannabis-using patients with SCZ show relatively preserved or even enhanced performance in domains such as Verbal Memory, Processing Speed, and Social Cognition [52,53,54]. Meta-analytic evidence also suggests that cannabis-using patients with psychosis may present with less severe cognitive deficits at the time of first episode, possibly reflecting differences in premorbid functioning [55].

Several hypotheses may account for this paradox. One possible explanation relates to neurodevelopmental and clinical subtypes of psychosis. Cannabis users with psychosis often display higher premorbid IQ, better social adjustment, and fewer negative symptoms than non-using patients [56,57]. Such characteristics suggest that cannabis-using patients may represent a distinct clinical phenotype with a relatively preserved neurodevelopmental profile. In our sample, the No-CU subgroup showed greater impairment in the MAP dimension of the BNSS, consistent with the observation that negative symptoms may be more severe in individuals who do not consume cannabis. Rather than reflecting a direct effect of cannabis use on negative symptoms or cognition, these differences may be more parsimoniously interpreted as indexing distinct clinical phenotypes within psychotic disorders. Specifically, patients characterized by a higher burden of negative symptoms, particularly motivational and hedonic deficits, appear less likely to engage in cannabis use, whereas those with relatively preserved motivation and reward processing may be more prone to substance use. Within this framework, the higher MAP scores observed in No-CU patients may reflect a negative-symptoms dominant phenotype, which is also associated with poorer cognitive performance [58], thereby contributing to the lower MoCA scores in this group. Notably, the No-CU group was also older than the CU group, a factor that may have contributed to the poorer cognitive performance.

Secondly, neuropharmacological mechanisms may further explain part of the clinical heterogeneity. Δ9-tetrahydrocannabinol (THC) modulates dopaminergic and glutamatergic neurotransmission through CB1 receptor activation. While chronic exposure is associated with neurotoxic and psychotogenic effects, acute THC administration may transiently enhance neural plasticity, cognitive flexibility, or social cognition [59,60]. Conversely, cannabidiol (CBD) has been shown to have anxiolytic, antipsychotic, and pro-cognitive properties, potentially mitigating THC’s detrimental impact [61,62]. Although precise CBD/THC ratios were not available for this study. In Albania, specifically, the only systematic evaluation available to date [63] reported THC concentrations ranging from approximately 1.07% to 12.13%, with CBD detected but not in proportions sufficient to counterbalance THC. More recent European reports suggest that illicit cannabis markets—including Albania’s—are increasingly dominated by high-THC, low-CBD profiles [64]. In contrast, the United Kingdom and other Western European countries have seen even higher THC concentrations and lower CBD/THC ratios in “high-potency” strains, profiles strongly linked to greater risk of cognitive impairment and psychotic exacerbation [30,65]. Conversely, strains with a more balanced or CBD-rich composition may exert protective or counterbalancing effects against THC-related neurotoxicity, potentially explaining part of the heterogeneity observed in cognitive outcomes among users with psychosis. Some studies suggest that cannabis might temporarily increase the efficiency of neural circuits involved in emotional regulation and social interactions, mitigating some of the functional impairments seen in psychotic disorders [53].

A further consideration involves the “self-medication hypothesis”. Some participants in our study reported using cannabis to alleviate distress, paranoia, or agitation. Although subjective relief should not be interpreted as therapeutic efficacy, it underscores the complex role cannabis plays in emotional and daily functioning. Cannabis may transiently alleviate negative effects or improve social engagement, which in turn may influence cognitive test performance.

Finally, timing and frequency of cannabis use likely modulate cognitive outcomes. Heavy and early-onset use during adolescence, when the prefrontal cortex and hippocampus are still developing, is consistently linked to more severe long-term cognitive deficits and heightened psychosis risk [12,66]. In contrast, adult-onset, particularly after the onset of psychotic symptoms, may have less detrimental effects, or in some cases appear neutral or paradoxically protective. Our results further showed that non-daily users performed better than non-users on the MoCA, suggesting that intermittent cannabis use may not reach the neurotoxic threshold observed in daily, heavy users. These findings align with dose-dependent models, where moderate or occasional use produces less harm than high-frequency consumption of high-potency cannabis [51].

### Limitations

This study involves several limitations. First, cognition was assessed using the MoCA, a brief screening tool that, while cost-effective and feasible in resource-limited settings, lacks the sensitivity to detect domain-specific deficits. Comprehensive neuropsychological batteries such as the MCCB or BACS would provide a more detailed characterization of cognitive profiles.

Second, observational design precludes causal inference, and unmeasured confounders such as severity of psychotic symptoms, comorbidities, concurrent substance use (e.g., alcohol, nicotine), detailed diagnostic stratification within the psychosis spectrum, and past medication adherence may have influenced outcomes. Moreover, the lack of longitudinal follow-up limits the ability to examine the temporal stability of the observed associations. Consequently, it was not possible to assess trajectories of illness, progression of cognitive impairment, or potential bidirectional relationships between cannabis use, symptom profiles, and cognition. Future longitudinal studies with repeated assessments are therefore warranted to clarify causality and better characterize developmental and illness-stage–related effects.

Another limitation is the absence of data distinguishing between THC- and CBD-dominant cannabis, which exert opposing effects on cognition and psychosis. A further limitation is that the frequency-based categorization of cannabis use represents only an approximate measure of exposure and may not fully capture interindividual variability in consumption patterns.

Finally, a major contextual limitation is the lack of recent data on the illicit market in Albania. Future studies should address this gap, as characterizing the THC/CBD ratio in locally consumed cannabis is crucial for a more accurate interpretation of paradoxical cognitive findings.

## 5. Conclusions

Our findings contribute to the growing body of literature on the complex and sometimes paradoxical relationship between cannabis use and cognition in individuals with psychotic disorders. While cannabis use is typically associated with detrimental cognitive outcomes and an increased risk of symptom exacerbation, the present study highlights that, in certain cases, patients with psychosis who use cannabis may demonstrate relatively preserved or even superior cognitive performance compared with non-using patients. These results raise important clinical and research questions. One interpretation is that cannabis-using patients may constitute a distinct subgroup within the broader psychosis spectrum, characterized by greater premorbid cognitive reserve, fewer negative symptoms, and better social adjustment [56,57]. Alternatively, neuropharmacological effects of cannabinoids—particularly the ratio of THC/CBD—may transiently modulate neural circuits underlying cognition, producing heterogeneous outcomes across patients [61,62]. Selection effects, whereby individuals with more severe cognitive impairment are less likely to use cannabis, may also play a role [55]. Disentangling this mechanism will require large-scale, longitudinal, and multimodal studies that integrate neuropsychological assessments with neuroimaging and biomarker data.

Clinically, these findings underscore the need for a nuanced approach. Any apparent cognitive “advantage” in cannabis-using patients must be carefully weighed against the well-documented risks of cannabis use, including increased relapse rates, poorer medication adherence, and, worse, long-term functional outcomes [51,67]. For clinicians, this paradox emphasizes the importance of individualized treatment planning, taking into account not only the cognitive but also the emotional and symptomatic effects of cannabis. Furthermore, the relationship between cannabis use, psychosis, and cognitive impairment, particularly among CHR-P individuals, is of crucial importance, given the widespread use of cannabis and the need to develop targeted cognitive interventions for this population [68]. Cannabis use is also particularly high among young people with neurodevelopmental disorders, and given the close link between neurodevelopmental conditions and psychosis risk, further studies are needed to clarify whether, and how, the interaction between cannabis use and cognitive functioning may influence the likelihood of transition to a psychotic disorder [69,70,71].

In conclusion, clarifying this relationship may ultimately support the refinement of early detection strategies, psychoeducation, and cognitive rehabilitation interventions tailored to cannabis-using patients with psychosis.

## Figures and Tables

**Table 1:** Sociodemographic and psychopathological characteristics of the sample.

Variables	Total Sample (*n* = 105; 100%)	CU (*n* = 56; 53.3%)	No-CU (*n* = 49; 46.7%)	Statistics ^a,b^	
				Value	*p*
Age	40.3 ± 11.84	34.8 ± 9.34	46.6 ± 11.3	569	**<0.001**
Years of education	12.8 ± 3.82	13.0 ± 3.81	12.5 ± 3.85	1272	0.495
Sex:				8.89	**0.004**
Females	34 (32.4%)	11 (10.5%)	23 (21.9%)		
Males	71 (67.6%)	45 (42.9%)	26 (24.8%)		
Years of illness	11.8 ± 8.1	8.52 ± 5.81	15.6 ± 8.66	693	**<0.001**
Number of hospital admissions	3.0 ± 1.8	3.04 ± 1.80	3.04 ± 1.86	1365	0.963
Use of antipsychotics:				2.60	0.122
Clozapine	27 (25.7%)	18 (17.1%)	9 (8.6%)		
Other antipsychotics	78 (74.3%)	38 (36.2%)	40 (38.1%)		
Long-acting injectable antipsychotics:				0.793	0.422
Yes	39 (37.1%)	23 (21.9%)	16 (15.2%)		
No	66 (62.9%)	33 (31.4%)	33 (31.4%)		
Diagnosis:				0.0321	0.858
non-affective psychosis	78 (74.3%)	42 (75%)	36 (73.5%)		
affective psychosis	27 (25.7%)	14 (25%)	13 (26.5%)		
Motivation-pleasure deficit (BNSS)	12.9 ± 7.06	11 ± 5.86	15 ± 7.74	975	**0.011**
Emotional expressivity deficit (BNSS)	4.42 ± 5.01	4.45 ± 4.95	4.39 ± 5.13	1334	0.805
CDSS	3.90 ± 3.14	3.29 ± 2.29	4.59 ± 3.80	1153	0.156
PSYRATS Delusion scale	16.5 ± 3.28	16.8 ± 3.35	16.3 ± 3.22	1237	0.384
PSYRATS Hallucination scale	22.9 ± 10.9	22.5 ± 9.91	23.3 ± 12.0	1228	0.354
TLC	17.8 ± 6.80	17 ± 6.14	18.7 ± 7.43	1185	0.230

^a^ Mann–Whitney U test, ^b^ chi-square (χ^2^) test. The chi-square *p*-values have been adjusted using Fisher’s exact test. Continuous variables are presented as means and standard deviations in years, and categorical variables are presented as counts and percentages. BNSS: Brief Negative Symptom Scale, CDSS: the Calgary Depression Scale for Schizophrenia, PSYRATS: the Psychotic Symptom Rating Scales; TLC: the Scale for the Assessment of Thought, Language, and Communication. Significant *p*-values are in bold.

**Table 2:** Cognitive differences between cannabis non-users (No-CU) and users (CU).

Variable	Z-Statistic ^a^	Raw *p*-Value	FDR *p*-Value
MoCA general score	−3.696	**0.0003**	**0.002400**
VisuoSpatial/Executive	−2.496	**0.0142**	**0.022720**
Naming	−0.289	0.7955	0.909143
Attention	−2.853	**0.0039**	**0.010400**
Language	−1.080	0.2629	0.350533
Abstraction	−3.214	**0.0006**	**0.002400**
Delayed Recall	−0.059	0.9723	0.972300
Orientation	−2.798	**0.0063**	**0.012600**

^a^ Mann–Whitney U test for the MoCa general index, and its sub-dimensions, between CU and No-CU. Significant *p*-values are in bold.

**Table 3:** Cognitive differences among non-users (No-CU), low (L-CU), and high frequency (H-CU) cannabis users.

Variable	H-Statistic ^a^	Raw *p*-Value	FDR *p*-Value
MoCA general score	15.051	**0.0003**	**0.002400**
VisuoSpatial/Executive	8.746	0.0102	0.026240
Naming	0.596	0.8061	0.806100
Attention	8.154	**0.0154**	**0.026240**
Language	1.285	0.5495	0.628000
Abstraction	11.419	**0.0029**	**0.011600**
Delayed Recall	1.600	0.4571	0.609467
Orientation	7.978	**0.0164**	**0.026240**

^a^ Kruskal–Wallis test for the MoCA general index, and its sub-dimensions, between the levels of the variable cannabis use. Significant *p*-values are in bold.

**Table 4:** Post hoc tests.

MoCA Variable	Comparison	Z-Statistic ^a^	*p*-Value ^b^
MoCA general score	No-CU vs. L-CU	4.23	**0.0073**
	No-CU vs. H-CU	4.62	**0.0026**
	L-CU vs. H-CU	1.75	0.4246
Visuospatial/Executive	No-CU vs. L-CU	2.40	0.2050
	No-CU vs. H-CU	3.97	**0.0122**
	L-CU vs. H-CU	2.32	0.2330
Attention	No-CU vs. L-CU	3.55	**0.0307**
	No-CU vs. H-CU	3.04	0.0802
	L-CU vs. H-CU	−0.08	0.9989
Abstraction	No-CU vs. L-CU	3.70	**0.0208**
	No-CU vs. H-CU	4.02	**0.0099**
	L-CU vs. H-CU	1.58	0.5039
Orientation	No-CU vs. L-CU	3.80	**0.0156**
	No-CU vs. H-CU	2.12	0.2904
	L-CU vs. H-CU	−0.71	0.8729
Naming	No-CU vs. L-CU	0.78	0.8144
	No-CU vs. H-CU	−0.46	0.9307
	L-CU vs. H-CU	−1.04	0.8144
Language	No-CU vs. L-CU	1.58	0.5213
	No-CU vs. H-CU	0.66	0.8976
	L-CU vs. H-CU	−0.52	0.9373
Delayed Recall	No-CU vs. L-CU	−0.63	0.8955
	No-CU vs. H-CU	1.54	0.5228
	L-CU vs. H-CU	1.65	0.4767

^a^ Wilcoxon rank-sum statistic; ^b^ Dwass–Steel–Critchlow–Fligner tests. Significant *p*-values are in bold.

## Data Availability

The data are not publicly available due to privacy or ethical restrictions.

## Supplementary Materials

The following supporting information can be downloaded at: https://www.mdpi.com/article/10.3390/pathophysiology33010011/s1, Figure S1: Distribution of MoCA subscales’ scores in cannabis users (CU) and non-users (No-CU); Figure S2: Distribution of MoCA subscales’ scores in non-users (No-CU), low-frequency users (L-CU), and high-frequency users (H-CU).

## Abbreviations

The following abbreviations are used in this manuscript:

AHS	Auditory Hallucinations Subscale
BACS	Brief Assessment of Cognition in Schizophrenia
BNA	Brief Neurocognitive Assessment
BNSS	Brief Negative Symptom Scale
CBD	Cannabidiol
CB1/CB2	Cannabinoid Receptor Type 1/Type 2
CHR-P	Clinical High Risk for Psychosis
CDSS	Calgary Depression Scale for Schizophrenia
CU	Cannabis Users
DS	Delusions Subscale
DSCF	Dwass–Steel–Critchlow–Fligner (test)
DSM-5	Diagnostic and Statistical Manual of Mental Disorders, Fifth Edition
EE	Emotional Expressivity
FDR	False Discovery Rate
FEP	First-Episode Psychosis
FTD	Formal Thought Disorder
H-CU	High-Frequency Cannabis Users
IQ	Intelligence Quotient
L-CU	Low-Frequency Cannabis Users
MAP	Motivation and Pleasure
MCCB	MATRICS Consensus Cognitive Battery
MCI	Mild Cognitive Impairment
MoCA	Montreal Cognitive Assessment
No-CU	Non-Cannabis Users
PLE	Psychotic-like Experiences
PSYRATS	Psychotic Symptom Rating Scales
SCIP	Screen for Cognitive Impairment in Psychiatry
SCZ	Schizophrenia
SD	Standard Deviation
TLC	Thought, Language, and Communication Scale
THC	Δ9-Tetrahydrocannabinol
WAIS-R	Wechsler Adult Intelligence Scale–Revised
