# Supplementary material for: The Paradoxical Effect of Cannabis Use on Cognition in Chronic Psychotic Disorders
Source: Pathophysiology. 2026 Jan 27;33(1):11. doi: 10.3390/pathophysiology33010011 (PMC12921724; doi:10.3390/pathophysiology33010011)
Supplement: Supplementary file 1 [file pathophysiology-33-00011-s001.zip › Supplementary materials_Figure S1.pdf]

Supplementary materials

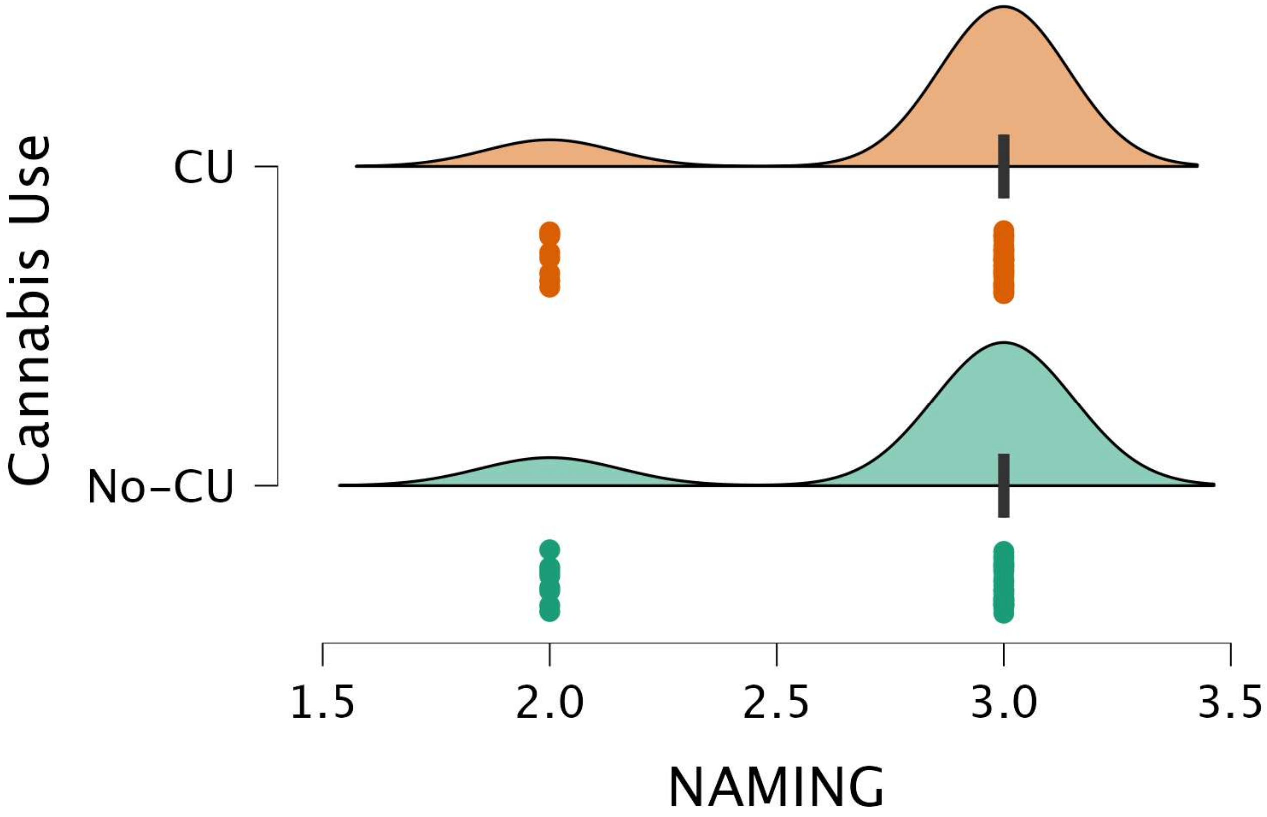

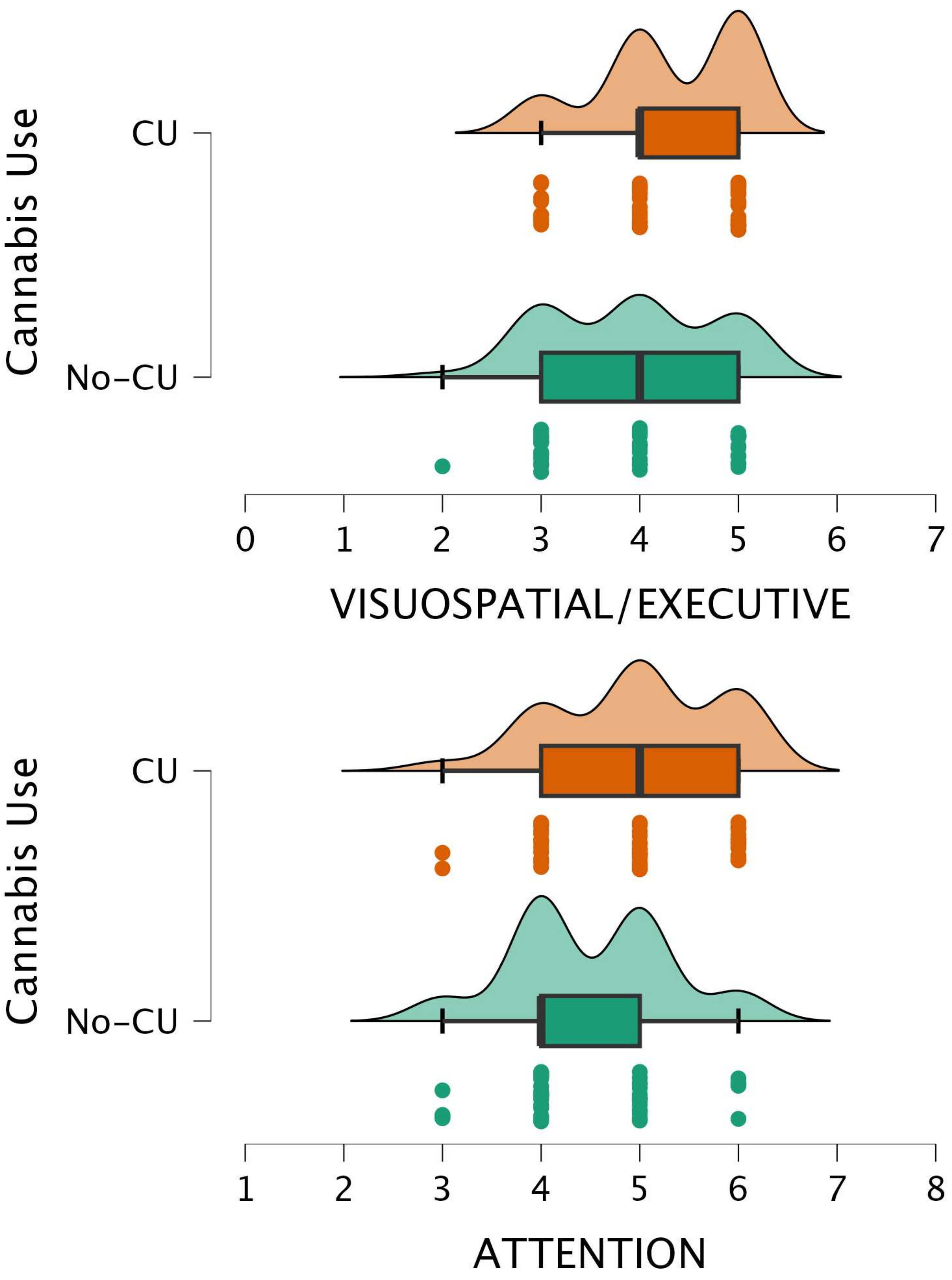
Cannabis Use
CU
No–CU
0
1
2
3
4
5
6
7
VISUOSPATIAL/EXECUTIVE
Cannabis Use
CU
No–CU
1
2
3
4
5
6
7
8
ATTENTION

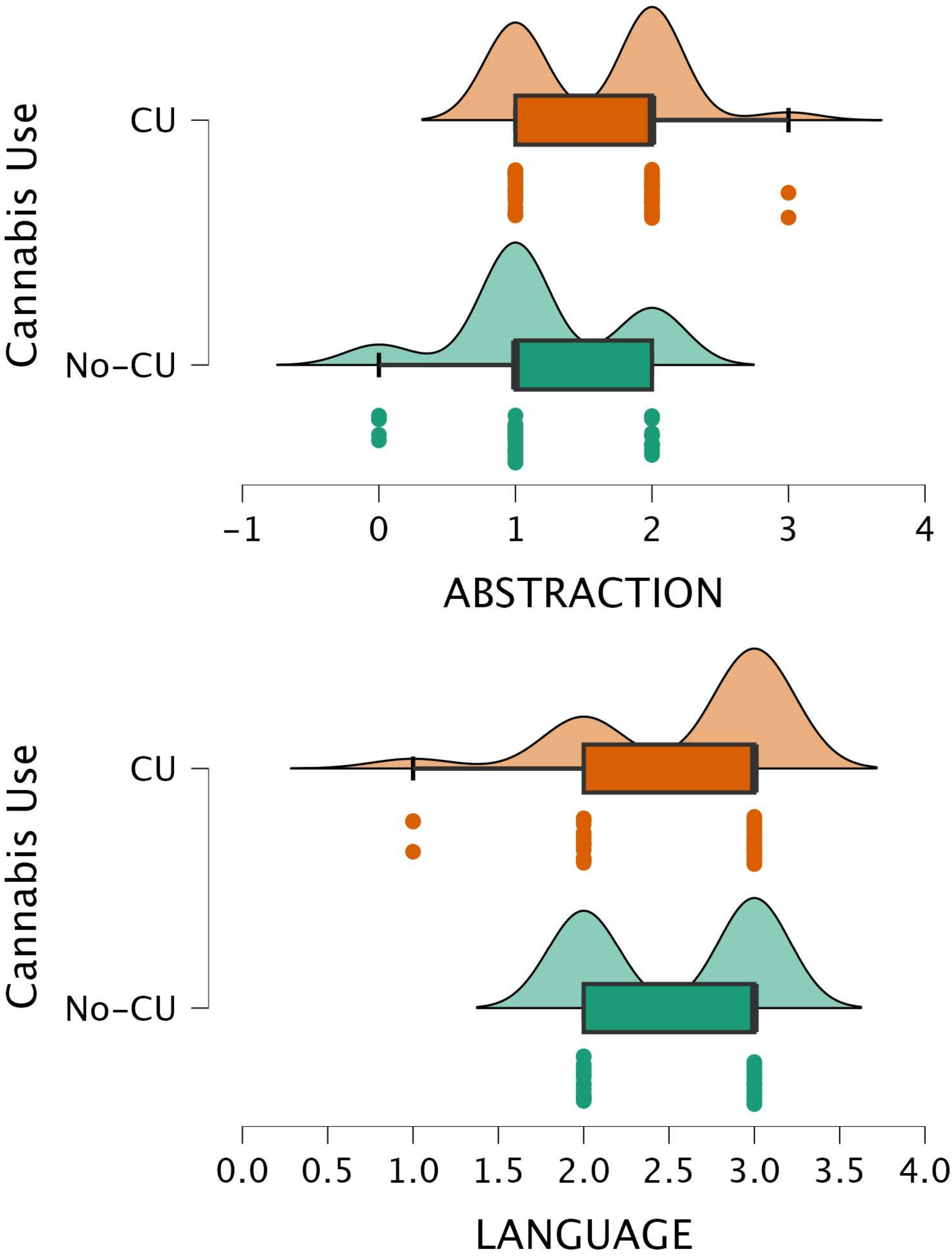

Cannabis Use
CU
No-CU
-1
0
1
2
3
4
ABSTRACTION
Cannabis Use
CU
No-CU
0.0
0.5
1.0
1.5
2.0
2.5
3.0
3.5
4.0
LANGUAGE

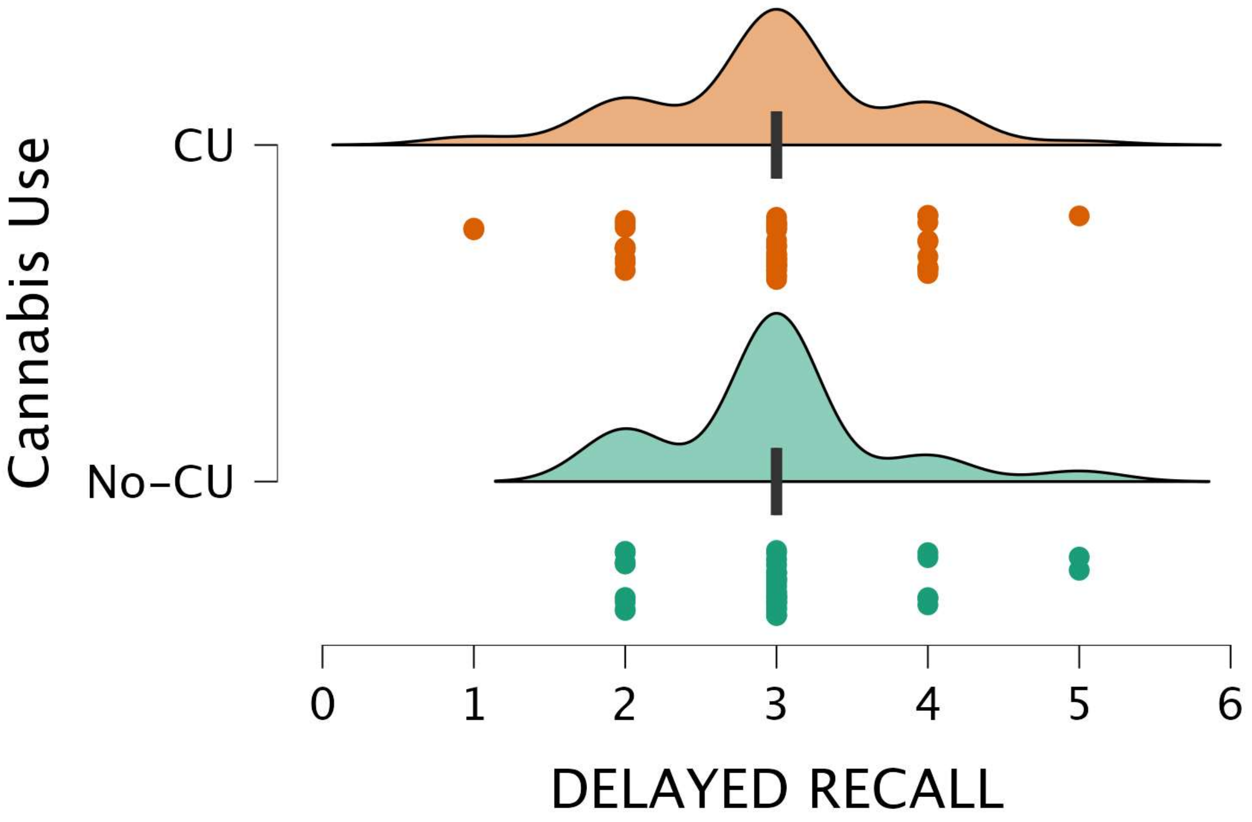

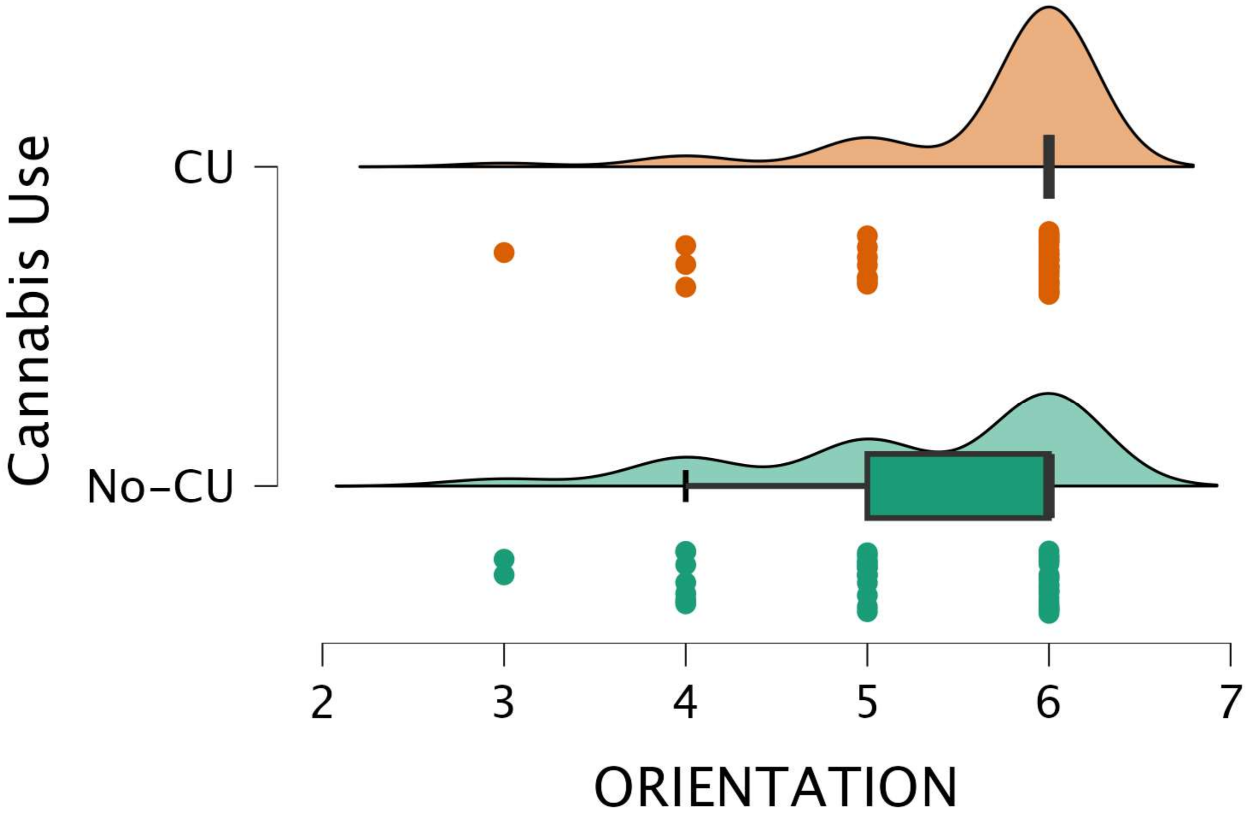

Figures S1 A-G
Distribution of MoCA subscales' scores in cannabis users (CU) and non-users (No-CU).
