# Supplementary material for: The Paradoxical Effect of Cannabis Use on Cognition in Chronic Psychotic Disorders
Source: Pathophysiology. 2026 Jan 27;33(1):11. doi: 10.3390/pathophysiology33010011 (PMC12921724; doi:10.3390/pathophysiology33010011)
Supplement: Supplementary file 1 [file pathophysiology-33-00011-s001.zip › Supplementary materials_Figure S2.pdf]

**Supplementary materials**

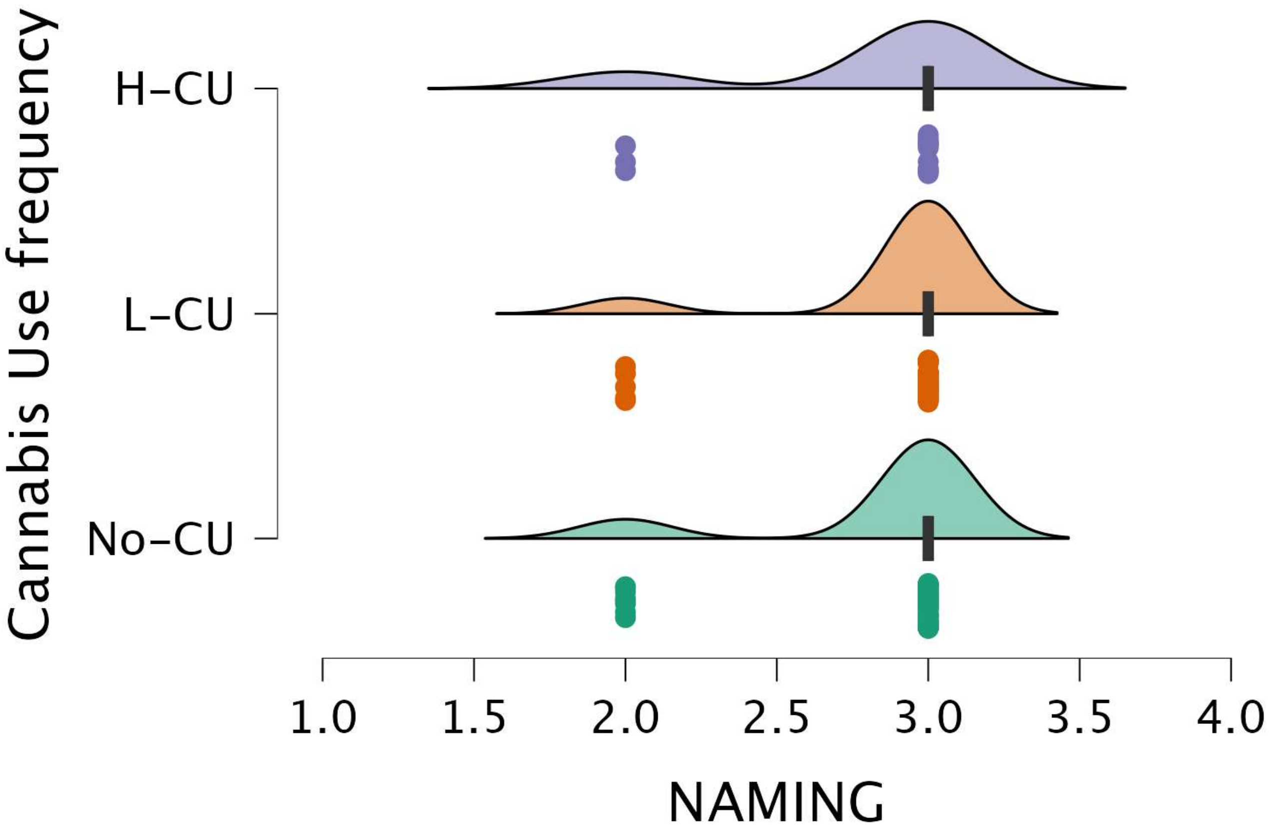

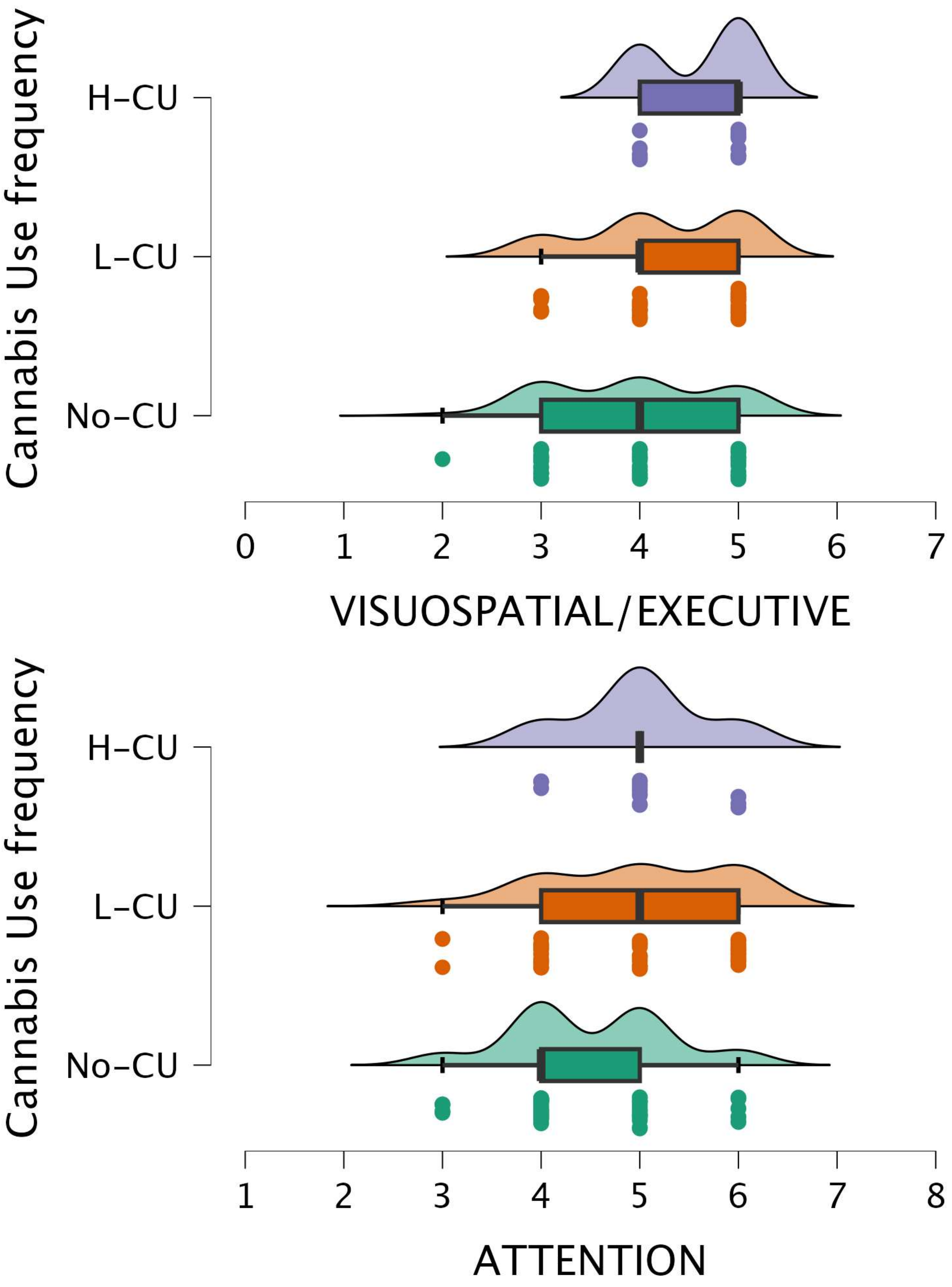
Cannabis Use frequency
H−CU
L−CU
No−CU
0
1
2
3
4
5
6
7
VISUOSPATIAL/EXECUTIVE
Cannabis Use frequency
H−CU
L−CU
No−CU
1
2
3
4
5
6
7
8
ATTENTION

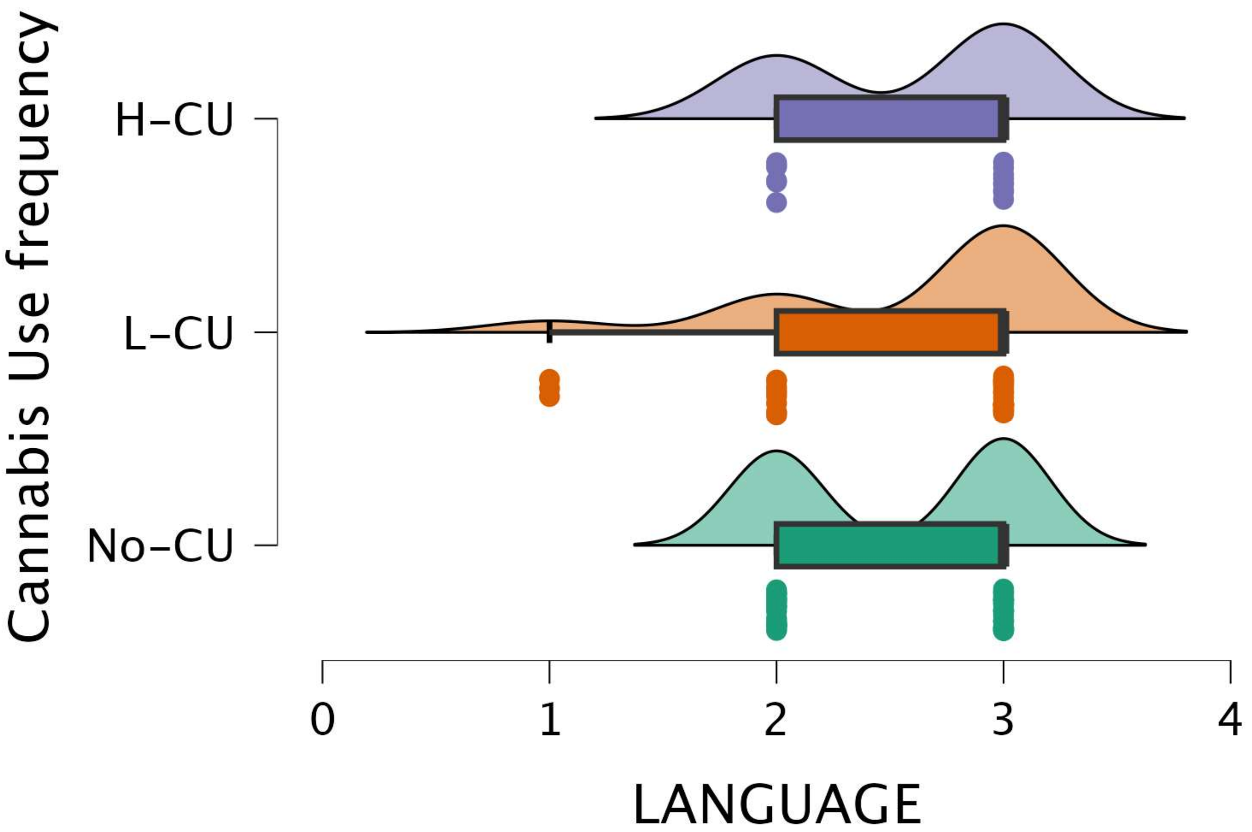

Cannabis Use frequency
H–CU
L–CU
No–CU
0
1
2
3
4
LANGUAGE

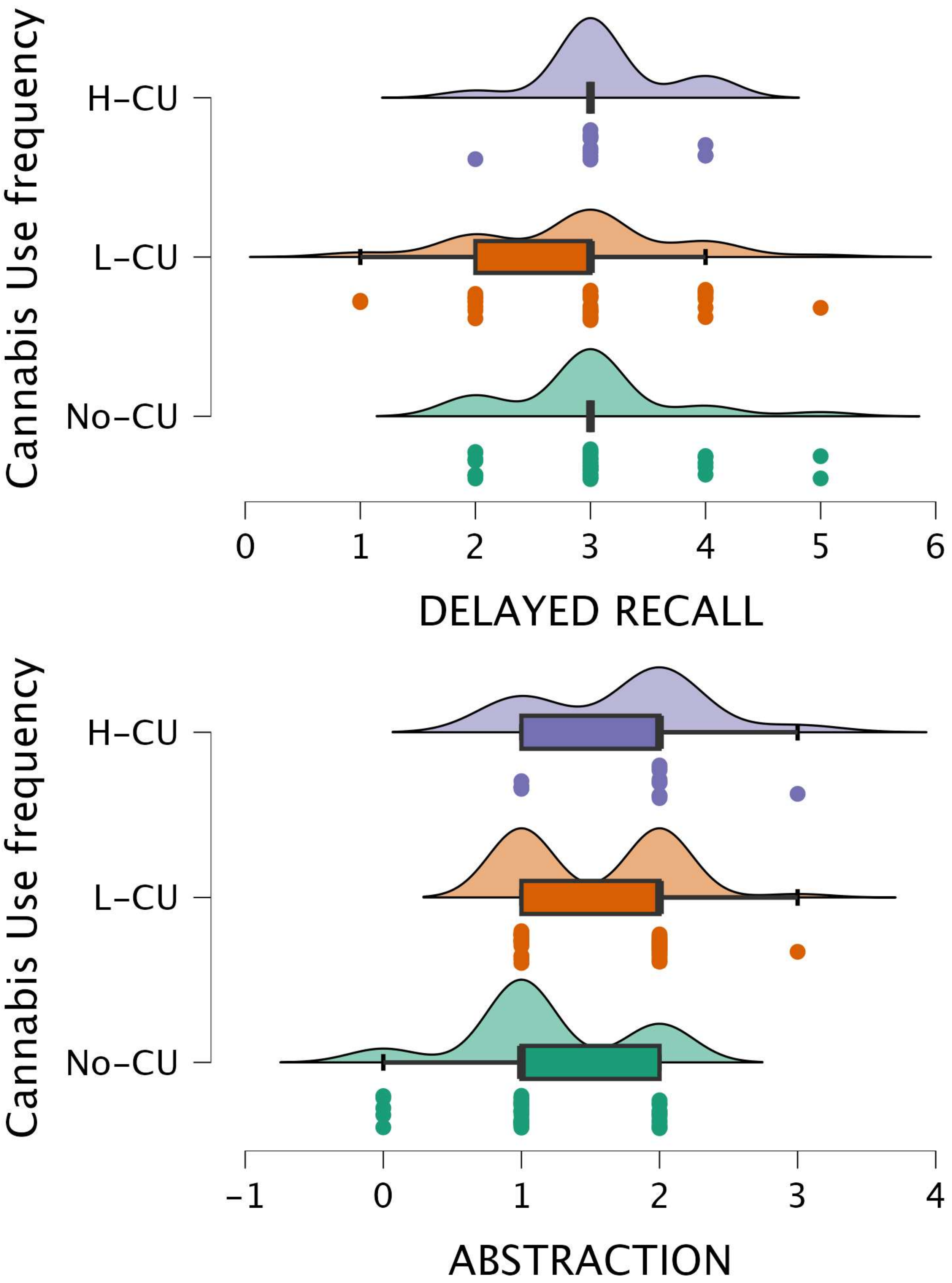
Cannabis Use frequency
H–CU
L–CU
No–CU
0
1
2
3
4
5
6
DELAYED RECALL
Cannabis Use frequency
H–CU
L–CU
No–CU
–1
0
1
2
3
4
ABSTRACTION

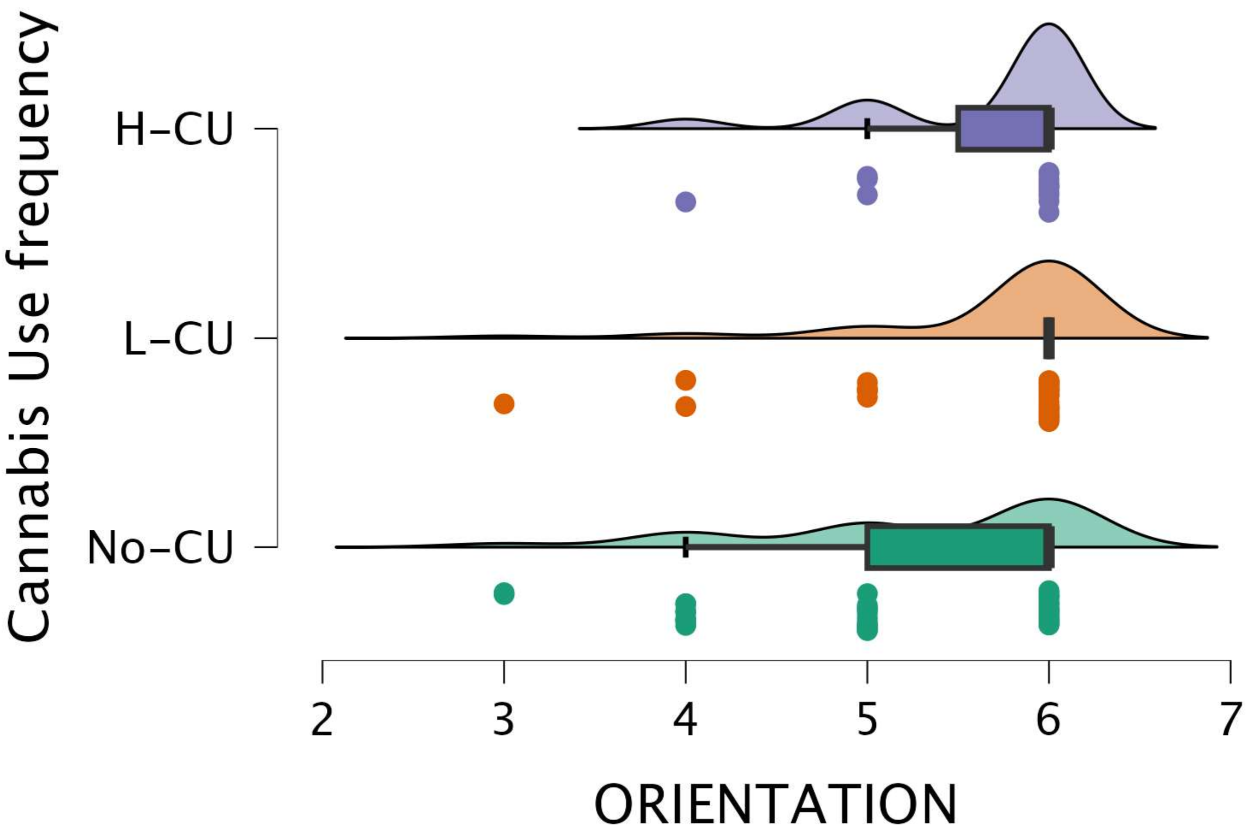


Figures S2 A-G
Distribution of MoCA subscales' scores in non-users (No-CU), low-frequency users (L-CU), and high-frequency users (H-CU).
